# Dietary quality and nutrient intake in adults with obsessive–compulsive disorder

**DOI:** 10.1192/bjo.2021.1039

**Published:** 2021-11-19

**Authors:** Thomas P. Nguyen, Lachlan Cribb, Chee H. Ng, Gerard J. Byrne, David Castle, Vlasios Brakoulias, Scott Blair-West, Georgina Oliver, Carolyn Ee, Olivia M. Dean, David A. Camfield, Chad Bousman, Nathan Dowling, Rajshri Roy, Michael Berk, Jerome Sarris

**Affiliations:** School of Medicine, Western Sydney University, Australia; and NICM Health Research Institute, Western Sydney University, Australia; Professorial Unit, The Melbourne Clinic, Department of Psychiatry, University of Melbourne, Australia; Professorial Unit, The Melbourne Clinic, Department of Psychiatry, University of Melbourne, Australia; University of Queensland Centre for Clinical Research, Royal Brisbane & Women's Hospital, Australia; and Mental Health Service, Royal Brisbane & Women's Hospital, Australia; Centre for Complex Interventions, Centre for Addictions and Mental Health, Toronto, Canada; and Department of Psychiatry, University of Toronto, Canada; School of Medicine, Western Sydney University, Australia; and Western Sydney Local Health District Mental Health Service, Australia; Professorial Unit, The Melbourne Clinic, Department of Psychiatry, University of Melbourne, Australia; Professorial Unit, The Melbourne Clinic, Department of Psychiatry, University of Melbourne, Australia; NICM Health Research Institute, Western Sydney University, Australia; IMPACT – the Institute for Mental and Physical Health and Clinical Translation, School of Medicine, Barwon Health, Deakin University, Australia; and Florey Institute for Neuroscience and Mental Health, Australia; IMPACT – the Institute for Mental and Physical Health and Clinical Translation, School of Medicine, Barwon Health, Deakin University, Australia; Department of Psychiatry, University of Melbourne, Australia; and Departments of Medical Genetics, Psychiatry, Physiology and Pharmacology, and Community Health Sciences, University of Calgary, Alberta, Canada; Professorial Unit, The Melbourne Clinic, Department of Psychiatry, University of Melbourne, Australia; Discipline of Nutrition and Dietetics, Faculty of Medical and Health Sciences, The University of Auckland, New Zealand; IMPACT – the Institute for Mental and Physical Health and Clinical Translation, School of Medicine, Barwon Health, Deakin University, Geelong, Australia; Department of Psychiatry, University of Melbourne, Australia; Florey Institute for Neuroscience and Mental Health, Melbourne, Australia; Orygen, The National Centre of Excellence in Youth Mental Health, Melbourne, Australia; and Centre for Youth Mental Health, University of Melbourne, Australia; NICM Health Research Institute, Western Sydney University, Australia; and Professorial Unit, The Melbourne Clinic, Department of Psychiatry, University of Melbourne, Australia

**Keywords:** Obsessive–compulsive disorder, dietary quality, nutrition, diet, psychiatry

## Abstract

**Background:**

Many mental disorders, including depression, bipolar disorder and schizophrenia, are associated with poor dietary quality and nutrient intake. There is, however, a deficit of research looking at the relationship between obsessive–compulsive disorder (OCD) severity, nutrient intake and dietary quality.

**Aims:**

This study aims to explore the relationship between OCD severity, nutrient intake and dietary quality.

**Method:**

A *post hoc* regression analysis was conducted with data combined from two separate clinical trials that included 85 adults with diagnosed OCD, using the Structured Clinical Interview for DSM-5. Nutrient intakes were calculated from the Dietary Questionnaire for Epidemiological Studies version 3.2, and dietary quality was scored with the Healthy Eating Index for Australian Adults – 2013.

**Results:**

Nutrient intake in the sample largely aligned with Australian dietary guidelines. Linear regression models adjusted for gender, age and total energy intake showed no significant associations between OCD severity, nutrient intake and dietary quality (all *P* > 0.05). However, OCD severity was inversely associated with caffeine (β = −15.50, 95% CI −28.88 to −2.11, *P* = 0.024) and magnesium (β = −6.63, 95% CI −12.72 to −0.53, *P* = 0.034) intake after adjusting for OCD treatment resistance.

**Conclusions:**

This study showed OCD severity had little effect on nutrient intake and dietary quality. Dietary quality scores were higher than prior studies with healthy samples, but limitations must be noted regarding comparability. Future studies employing larger sample sizes, control groups and more accurate dietary intake measures will further elucidate the relationship between nutrient intake and dietary quality in patients with OCD.

Obsessive–compulsive disorder (OCD) is a debilitating and disabling mental illness that is complicated by its psychiatric comorbidities (e.g. major depressive disorder, obsessive–compulsive personality disorder and generalised anxiety disorder) and significant functional impairment.^1^ Clinical guidelines recommend cognitive–behavioural therapy, exposure and response prevention and selective serotonin reuptake inhibitors as first-line treatments for OCD.^2,3^ However, symptom improvement from these treatments may take weeks to months, and up to 40–60% of patients are treatment-resistant to selective serotonin reuptake inhibitors.^2,3^ Given the existing limitations of first-line treatments for OCD, a primary preventative approach that targets the modifiable risk factors of mental illness must also be considered.

## Mechanisms implicated in diet and OCD

Advances in developing novel therapies for OCD have been limited by an imprecise understanding of its specific pathophysiology. However, high levels of oxidative stress, cortisol, inflammation, gut microbial dysfunction and genetic mutations related to mitochondrial dysfunction have been suggested as potential mechanisms relevant to OCD pathology.^4–6^

Many of these proposed mechanisms may be modified by dietary quality or dietary intake. Vitamins and polyphenols in plant-based foods, as well as extra virgin olive oil and omega-3 in fish, have been shown to exert anti-inflammatory effects.^7^ Conversely, calorie-dense foods, hydrogenated fats and added sugars are linked with the release of pro-inflammatory cytokines.^7^ Foods with high fibre, prebiotics and probiotics may modulate gut microbiota, whereas Mediterranean and other plant-rich diets have been linked with increased microbiota diversity.^7^ With respect to mitochondrial dysfunction, preclinical evidence suggests that high-fat diets may be associated with abnormal biogenesis of mitochondria, which is linked with greater free radical production.^8^

Meta-analyses of randomised controlled trials (RCTs) and observational studies have found that improving dietary quality results in significant reductions in depressive symptoms, especially when interventions are delivered by health professionals.^9,10^ However, it must be noted that most of these studies used samples with non-clinical depression and significant heterogeneity was encountered in subgroup analyses.^9,10^ Given considerations such as these, recommendations for modifying diet as part of managing mental illness have begun receiving international recognition in several national health policy documents and psychiatric clinical guidelines.^11^

## Diet and OCD

Dietary and nutritional concerns in OCD may be additionally important given the increased risk of chronic disease in patients with OCD. Increased risk of metabolic and cardiovascular disease, which are substantially influenced by dietary habits, have been reported in individuals with OCD.^12^ Furthermore, an Italian cross-sectional study of 104 patients with OCD found that the prevalence of metabolic syndrome was higher than the general population, with its risk increasing with prolonged antipsychotic use.^13^ At the physiological level, a study comparing 104 patients with severe OCD symptoms and 101 patients admitted to a psychiatric unit (with predominantly psychotic and mood disorders) found that the patients with OCD had significantly higher levels of blood cholesterol and creatinine, despite being on lower doses of antipsychotics.^14^ Although it is well-established that individuals with depression, bipolar disorder and psychotic disorders often have poor dietary quality, no published study to date has investigated the overall dietary quality of individuals with OCD.^8,15^

Studies have reported that the serum levels of certain micronutrients, such as serum vitamin B12, zinc, iron and magnesium, are lower in people with OCD compared with healthy controls, although the relationship with folic acid or folate with OCD has been less conclusive.^16–20^ Moreover, emerging evidence suggests that zinc and magnesium may have a significant and negative correlation with depressive symptoms in women, but not men, although clinical samples are also required.^21–23^ Lastly, a small (*n* = 11) RCT found augmenting fluoxetine (20 mg/day) with zinc sulphate (440 mg/day) achieved significant decreases in Yale Brown Obsessive–Compulsive Scale (Y-BOCS) scores after 8 weeks.^24^ Although this emerging research is promising, larger sample sizes with more robust study designs are needed to elucidate the importance of these micronutrients in OCD.

As little is known about the overall dietary quality and macro and micronutrient intake in patients with OCD, the present study evaluated the nutrient intake and dietary quality in adults with OCD by using a standardised dietary quality assessment. Regression analyses were employed to determine whether the severity of OCD symptoms correlated with dietary quality and nutrient intake. Given the emerging data suggesting that patients with OCD have impaired markers of physical health, we hypothesised that nutrient intake and dietary quality would be worse in patients with OCD compared with the general population. Additionally, consistent with evidence in studies of depression symptoms,^21–23^ we hypothesised that there would be a negative relationship between OCD severity and zinc and magnesium intake in women, but not men.

## Method

### Study overview, design and procedure

Baseline data were drawn from participants with DSM-5-diagnosed OCD in two separate studies, and were used to investigate the relationship between OCD severity, nutrient intake and dietary quality. The first study (*n* = 98) was a phase 3, multicentre, 24-week, randomised, double blind placebo-controlled trial studying the effects of N-acetylcysteine (NAC) augmentation (2–4 g/day) compared with placebo in the treatment of OCD.^25^ Participants were recruited between 2016 and 2020 at The Melbourne Clinic, Melbourne (University of Melbourne); Royal Brisbane and Women's Hospital, Brisbane (University of Queensland); and NICM Health Research Institute, Westmead (Western Sydney University). Eligibility criteria included being aged 18–75 years, capacity to consent to the study and follow its procedures, primary DSM-5 diagnosis of OCD and a score of 16–31 on the Y-BOCS. Participants were excluded if they had bipolar disorder, a psychotic disorder, a primary diagnosis of obsessive–compulsive spectrum disorder, severe depression, alcohol/substance misuse, treatment refractory OCD, were currently engaging in intensive psychological therapies for OCD or were taking medications with known or suspected negative interactions with NAC (e.g. activated charcoal, nitroglycerine, chloroquine). Other exclusion criteria included allergy to any component of the investigational product, serious and/or unstable medical conditions, recent gastrointestinal ulcers, pregnancy and lactation, participation in any other interventional study and cessation of primary OCD medication. Participants were also required to remain on the same dose of psychotropic medication/s for their OCD throughout the study period.

The second study (*n* = 28) was a 20-week, open-label pilot study that assessed the effectiveness and safety of a nutraceutical formulation for patients with treatment-resistant DSM-5-diagnosed OCD.^26^ Participants were recruited at the same sites as the first study described above, between 2017 and 2020. Eligibility criteria included being aged 18–75 years, capacity as well as desire to consent to the study and follow its procedures, a primary DSM-5 diagnosis of moderate-to-extreme OCD (Y-BOCS ≥ 16), inadequate responses to at least three trials of serotonin reuptake inhibitors and at least one augmentation strategy as well as engagement in adequate OCD-specific cognitive–behavioural therapy. Participants were excluded if they had bipolar disorder, a psychotic disorder, a primary diagnosis of obsessive–compulsive spectrum disorders, severe depression, alcohol/substance misuse, suicidal ideation (defined as Structured Interview Guide for the Hamilton Rating Scale for Depression (SIGH-D) item score ≥3), serious and/or unstable medical conditions or allergies to the nutraceuticals studied (NAC, L-theanine, zinc, selenium, magnesium and pyridoxal-5’-phosphate).

Participants in both studies were recruited via invitation letters to former attendees of an OCD in-patient programme at The Melbourne Clinic, clinician referrals at all three recruitment sites, as well as radio and social media advertisements. Those who were interested in the study completed a brief phone screening assessment with a trained research assistant to assess study suitability; baseline screening appointments were subsequently scheduled for those who were eligible. Following written informed consent, participants were assessed by a trained research assistant at baseline, using the measures outlined below. Both studies used the Structured Clinical Interview for the DSM-5 to confirm OCD diagnoses. The Y-BOCS and SIGH-D were also administered at baseline. These data were used in the current subanalysis. Participants were asked to complete the Dietary Questionnaire for Epidemiological Studies version 3.2 at home. However, of the 126 participants, a large proportion (*n* = 41) did not complete the survey, as it was not a compulsory component of the trial.

### Measures

#### Y-BOCS

The Y-BOCS consists of the Yale Brown Obsessive–Compulsive Scale-Severity Scale (Y-BOCS-SS) and the Yale Brown Obsessive–Compulsive Scale-Symptom Checklist.^27,28^ The Y-BOCS-SS was the primary outcome measure in both parent trials.

The Y-BOCS-SS is a ten-item clinician-administered instrument that rates the severity of obsessive and compulsive symptoms (e.g. time spent on obsessions) on a five-point Likert scale from 0 (none or minimal severity) to 4 (greatest severity).^28^ The sum of these ten items allows an individual's OCD to be categorised as either subclinical (0–7), mild (8–15), moderate (16–23), severe (24–31) or extreme (32–40). The Y-BOCS-SS demonstrates good psychometric properties and is considered to be the gold standard in assessing OCD symptom severity.^29^

#### DQES version 3.2

The DQES version 3.2 is an online self-administered food frequency questionnaire (FFQ) developed by the Cancer Council Victoria, Australia. It was developed and validated to assess dietary and nutritional intake in Australian adults.^30^ The DQES version 3.2 consists of 142 food and beverage items and provides estimates of 98 nutrient indices, including macronutrients, micronutrients, glycaemic index, fibre and alcohol.^31^

#### Healthy Eating Index for Australian Adults – 2013

The Healthy Eating Index for Australian Adults – 2013 (HEIFA-2013) is a gender-specific index for dietary quality that assesses adherence to serving size recommendations outlined in the 2013 Australian Dietary Guidelines.^32^ The index consists of 11 components, nine of which are scored from zero to ten (i.e. discretionary foods, vegetables, fruits, grains, meat and its alternatives, dairy and its alternatives, saturated fat, sodium, added sugar) and two of which are scored from zero to five (i.e. water and alcohol intake).^32^ Higher scores reflect intake of a certain food group that more closely matches the recommended serving size, with the highest possible total score being 100.^32^ The HEIFA-2013 has been tested for criterion validity and internal consistency and has been used numerous times in adult populations.^32,33^ In this study, nutrient intakes as measured by the DQES version 3.2 were used to score each participant's dietary quality.

#### Other measures

Other measures included the Dimensional Obsessive–Compulsive Scale (DOCS), 17-item SIGH-D, Beck Anxiety Inventory (BAI), Sheehan Disability Scale, Clinical Global Impression Scale (CGIS) and World Health Organization-Quality of Life-BREF. Blood pressure and body mass index measurements were also taken at baseline.

### Ethics approval and consent to participate

The first trial received ethical clearance through the Human Research Ethics Committees (HREC) of The Melbourne Clinic Research Ethics Committee (HREC number 279), The University of Queensland Medical Research Ethics Committee (HREC number 2016001720) and Western Sydney University Human Research Ethics Committee (HREC number H12181). The second trial received ethical clearance through The Melbourne Clinic Research Ethics Committee (HREC number 290), The University of Queensland Medical Research Ethics Committee (HREC number 2018000339) and Western Sydney University Human Research Ethics Committee (HREC number H12331). All components of both parent trials were carried out in line with the latest version of the Declaration of Helsinki and were preregistered on the Australian New Zealand Clinical Trials Registry (identifiers ACTRN12616000847415 and ACTRN12617001140347, respectively). Written and informed consent was provided by a signature to the consent form after a trained research assistant explained the study to the participant.

### Statistical analyses

Sociodemographic characteristics were compared between the groups that completed and did not complete the DQES version 3.2, using Mann–Whitney U tests for continuous variables and chi-squared tests for categorical variables. For certain mental health scales (e.g. DOCS, BAI), participants occasionally missed items when completing the questionnaire. Missed items were imputed with predictive mean matching (R package ‘mice’).^34^ Under 5% of items were imputed.

The relationship between OCD severity, dietary quality and nutrient intake was assessed with linear regression. Model 1 assessed whether greater OCD severity was related to a greater intake of a given nutrient, adjusting for gender, age and energy intake. Sensitivity analyses were also performed with depression (using the Hamilton Rating Scale for Depression) and treatment resistance included as covariates to determine whether OCD severity was associated with nutrient intakes after accounting for depression symptoms and other covariates (Supplementary Material available at https://doi.org/10.1192/bjo.2021.1039). Assumptions of linear regression were checked via residual versus fitted plots.

The HEIFA-2013 scores were calculated with Microsoft Excel 365 for Windows, and all other statistical analyses were conducted on IBM SPSS Statistics 27 for Windows and R version 4.0.4 for Windows. Statistical significance was regarded as *P* < 0.05.

## Results

### Participant characteristics and psychological features

Of the 126 participants across both parent studies, 85 completed the DQES version 3.2 and were hence included in this substudy. Sociodemographic characteristics are shown in Table 1. Participants who did not complete the DQES version 3.2 reported significantly higher total Y-BOCS (mean_no-DQES_ = 24.53 *v*. mean_DQES_ = 22.73, *P* = 0.025) and CGIS (mean_no-DQES_ = 4.39 *v*. mean_DQES_ = 4.13, *P* = 0.034) scores than those who completed the DQES. However, there were no statistically significant differences for any of the other sociodemographic characteristics and psychological features assessed, such as gender, age and educational status. Of the 85 participants, the mean age was 36.95 years (s.d. = 14.3), 67.1% were women, and the mean body mass index was 26.94 (s.d. = 6.3).

**Table 1:** Participant characteristics

Characteristic	Summary
Age, years, mean (s.d.)	36.95 (14.3)
Gender, *n* (%)	
Male	28 (32.9)
Female	57 (67.1)
Body mass index, mean (s.d.)	27.14 (6.3)
Marital status, *n* (%)	
Single	42 (49.4)
Married/*de facto*	32 (37.6)
Separated/divorced/widowed	7 (28.2)
In a relationship	4 (4.7)
Education, *n* (%)	
High school graduate or early leaver	29 (34.1)
College certificate/diploma	18 (21.2)
University graduate/postgraduate	38 (44.7)
Employment status, *n* (%)	
Employed	46 (54.1)
Student	14 (16.5)
Unemployed	25 (29.4)
Trial, *n* (%)	
NAC	67 (78.8)
TRON	18 (21.2)
Trial site, *n* (%)	
Melbourne	43 (50.6)
Brisbane	30 (35.3)
Sydney	12 (14.1)

NAC, N-acetylcysteine; TRON, Treatment of Refractory Obsessive–Compulsive Disorder with Nutraceuticals.

Table 2 reports the main psychological features of the 85 participants. The mean age of OCD symptom onset was 13.4 years, with the mean age of OCD diagnosis being 23.8 years. Participants on average reported OCD symptoms for 23.3 years and having a diagnosis of OCD for an average of 13.1 years. Participants had on average 2.6 unsuccessful medication and therapy trials, and 21.2% of all participants were treatment-resistant.

**Table 2:** Participant psychological features

Psychological characteristic	Summary
Age at onset of OCD symptoms, years, mean (s.d.)^a^	13.4 (9.8)
Duration of OCD symptoms, years, mean (s.d.)^a^	23.3 (12.2)
Age of OCD diagnosis, years, mean (s.d.)	23.85 (10.8)
Chronicity of OCD diagnosis, years, mean (s.d.)	13.15 (10.7)
Number of unsuccessful prior medication and therapy trials, mean (s.d.)^a^	2.66 (2.9)
Treatment resistance, *n* (%)	
Yes	18 (21.2)
No	67 (78.8)
Y-BOCS total, mean (s.d.)	22.7 (4.5)
Moderate, *n* (%)	52 (61.2)
Severe, *n* (%)	31 (36.5)
Extreme, *n* (%)	2 (2.4)
DOCS total, mean (s.d.)^b^	29.9 (13.2)
BAI total, mean (s.d.)	18.1 (11.8)
SIGH-D total, mean (s.d.)	10.0 (6.3)
SDS total, mean (s.d.)	13.4 (6.9)
WHOQOL-BREF total, mean (s.d.)^c^	80.6 (13.5)
Patient Global Impression Scale, mean (s.d.)^d^	2.9 (0.6)
Clinical Global Impression Scale, mean (s.d.)	4.1 (0.8)
Current major depressive episode, *n* (%)	14 (16.5)
Previous major depressive episodes, *n* (%)^a^	53 (62.4)
Diagnosis of generalised anxiety disorder, *n* (%)^c^	19 (23.2)
Diagnosis of panic disorder, *n* (%)^e^	13 (16)
Diagnosis of social anxiety disorder, *n* (%)^f^	15 (19)
Diagnosis of excoriation disorder, *n* (%)^e^	8 (9.9)

OCD, obsessive–compulsive disorder; Y-BOCS, Yale–Brown Obsessive–Compulsive Scale; DOCS, Dimensional Obsessive–Compulsive Scale; BAI, Beck Anxiety Inventory; SIGH-D, Structured Interview Guide for the Hamilton Depression Rating Scale; SDS, Sheehan Disability Scale; WHOQOL-BREF, World Health Organization Quality of Life **–** BREF.

a. *n* = 2 missing.

b. *n* = 8 missing.

c. *n* = 3 missing.

d. *n* = 1 missing.

e. *n* = 4 missing.

f. *n* = 6 missing.

From the Y-BOCS scores, over half (61.2%) reported moderate OCD symptoms, 36.5% reported severe OCD symptoms, whereas 2.4% reported extreme OCD symptoms. The mean DOCS score (29.9) was above the minimum that is suggestive of a diagnosis of OCD.^18–20^ The mean BAI score (18.1) suggests that participants were moderately anxious on average, with 22.4% and 14.1% of the sample reporting a diagnosis of generalised anxiety disorder and panic disorder, respectively. Over half (62.4%) of the sample reported a previous major depressive episode, with 16.5% experiencing current clinical depression.

### Participant dietary intake and quality

Table 3 shows the mean macronutrient and micronutrient dietary intakes in this sample by total and gender, as well as the HEIFA-2013 dietary quality scores as summed by the individual food products in the DQES version 3.2. Estimates for the daily recommended intake in those aged 31–50 years are also outlined where possible, as per Australian guidelines.^35,36^ The mean energy intake was 8297 kJ/day (s.d. = 2628.1). Men consumed 9439.47 kJ/day (s.d. = 2697.85), which is greater than the recommended 8700 kJ average energy intake level that is commonly displayed on Australian nutrition information panels.^37^ Most participants (90.6%) ate the recommended daily serves of unsaturated fat (four serves for men, two serves for women). However, most participants (60%) consumed >10% of saturated fat as part of their total daily energy intake (mean 12.6%). Both men and women consumed more magnesium than the upper limit suggested (350 mg/day), although exceeding this value has not been shown to produce adverse effects.^29^ The mean sodium intake was 2172.13 mg (s.d. = 805.5); however, men consumed 2556.05 mg/day (s.d. = 920.82), which is above the recommended maximum of 2300 mg/day.^35^ The mean HEIFA-2013 score for dietary quality was 59.9 (s.d. = 13.7).

**Table 3:** Participant dietary intake and quality with Australian recommended intake levels

Dietary characteristic	Overall, mean (s.d.)	Men, mean (s.d.)	Women, mean (s.d.)	Upper limit	Recommended intake for those aged 31–50 years^a^	
					Men	Women
Energy, kJ/day	8297.94 (2628.1)	9439.47 (2697.85)	7737.18 (2424.71)	–	8700 (dependent on body mass index, physical activity level and age)	
Protein, g/day	84.78 (32.1)	99.07 (33.84)	77.76 (29.05)	–	64	46
Fat, g/day	84.16 (27.5)	92.64 (29.77)	80.00 (25.55)	–	–	–
Saturated fatty acids, g/day	27.83 (10.8)	31.33 (10.83)	26.11 (10.40)	–	–	–
Monounsaturated fatty acids, g/day	35.46 (12.1)	39.34 (13.86)	33.55 (10.69)	–	–	–
Polyunsaturated fatty acids, g/day	14.93 (6.3)	15.71 (6.72)	14.54 (6.03)	–	13^b^	8^b^
Long-chain omega 3 fatty acids, mg/day	343.16 (313.2)	449.17 (363.76)	291.09 (273.86)	–	–	–
Eicosapentaenoic acid, mg/day	88.26 (84.1)	119.33 (102.75)	72.99 (69.36)	–	–	–
Docosahexaenoic acid, mg/day	176.70 (183.1)	244.04 (232.65)	143.62 (144.30)	–	–	–
Carbohydrates, g/day	189.06 (67.2)	214.92 (65.99)	176.35 (64.63)	–	–	–
Sugar, g/day	95.31 (37.6)	106.91 (33.54)	89.61 (38.44)	–	–	–
Alcohol, g/day	7.93 (14.2)	10.79 (13.58)	6.52 (14.44)	Men: 40, women: 20	–	–
Fibre, g/day	25.43 (11.8)	26.24 (13.23)	25.03 (11.10)	–	30^c^	25^c^
Caffeine, mg/day	278.28 (278.8)	366.66 (353.33)	234.86 (224.92)	400	Not applicable	Not applicable
Water, g/day	4924.78 (1059.7)	3333.69 (1103.52)	2849.84 (1009.44)	–	3400^c^	2800^c^
Cholesterol, mg/day	274.36 (124.3)	301.19 (114.21)	261.17 (127.91)	–	–	–
Calcium, mg/day	865.88 (372.2)	1001.66 (353.15)	799.33 (366.01)	2500	1000	1000
Folate, μg/day)	420.41 (194.1)	446.06 (221.50)	407.81 (179.83)	1000	400	400
Iron, mg/day	10.96 (3.8)	11.69 (4.46)	10.61 (3.39)	45	8	18
Magnesium, mg/day	430.2 (164.7)	493.00 (165.96)	399.28 (156.43)	350	420	320
Sodium, mg/day	2172.13 (805.5)	2556.05 (920.82)	1983.55 (674.27)	2300	460–920^c^	460–620^c^
Vitamin B12, mg/day	3.13 (1.7)	4.20 (1.9)	2.61 (1.37)	80	2.4	2.4
Vitamin D, μg/day	3.72 (2.6)	5.19 (2.9)	3.00 (2.11)	80	5^c^	5^c^
Zinc, mg/day	8.84 (3.8)	9.96 (4.3)	8.29 (3.34)	40	14	8
HEIFA-2013 total	59.9 (13.7)	56.2 (14.5)	58.4 (12.2)	100	–	–
Discretionary	5.2 (3.9)	4.6 (4.2)	5.5 (3.7)	10	–	–
Vegetables	4.6 (2.8)	3.7 (2.7)	5.1 (2.8)	10	–	–
Fruit	8.0 (1.9)	7.8 (2.3)	8.1 (1.7)	10	–	–
Grains	3.9 (3.1)	5.0 (3.4)	3.5 (2.9)	10	–	–
Protein	7.9 (2.9)	7.7 (2.9)	8.1 (2.9)	10	–	–
Dairy	5.2 (3.5)	7.1 (3.3)	4.3 (3.2)	10	–	–
Fluid	3.6 (1.6)	3.3 (1.7)	3.7 (1.5)	5	–	–
Fat	6.3 (2.0)	6.3 (2.1)	6.4 (1.9)	10	–	–
Sodium	4.5 (4.0)	3.2 (3.7)	5.1 (4.1)	10	–	–
Sugar	6.3 (4.2)	5.7 (4.5)	6.6 (4.0)	10	–	–
Alcohol	4.3 (1.8)	3.8 (2.2)	4.6 (1.4)	5	–	–

HEIFA-2013, Healthy Eating Index for Australian Adults – 2013.

a. Recommended daily intake unless otherwise stated.

b. Linoleic acid; recommended daily intakes are taken from the Nutrient Reference Values for Australian and New Zealand.

c. Adequate intake.

### Associations between dietary intake/quality and total Y-BOCS score

Table 4 shows the associations between total Y-BOCS score, nutrient intake and dietary quality, using adjusted regression analyses. There was some weak (albeit non-significant) evidence suggesting that a greater Y-BOCS score was associated with a lower intake of alcohol (β = −0.58 g/day, 95% CI −1.26 to 0.10, *P* = 0.09), caffeine (β = −12.99 mg/day, 95% CI −26.07 to 0.09, *P* = 0.051) and magnesium (β = −5.46, 95% CI −11.42 to 0.50, *P* = 0.07) after accounting for gender, age and total energy intake. There was no clear evidence that OCD severity was associated with the intakes of any of the other assessed nutrients. Furthermore, the total Y-BOCS score was not associated with dietary quality scores (β = 0.06, 95% CI −0.58 to 0.70, *P* = 0.85).

**Table 4:** Associations between total Y-BOCS score and dietary intake/quality

Dietary characteristic	Model 1^a^	
	Adjusted coefficient (95% CI)^b^	*P*-value
Protein, g/day	0.35 (−0.43 to 1.13)	0.37
Fat, g/day	0.45 (−0.16 to 1.06)	0.15
Saturated fatty acids, g/day	0.22 (−0.07 to 0.50)	0.14
Monounsaturated fatty acids, g/day	0.14 (−0.22 to 0.51)	0.43
Polyunsaturated fatty acids, g/day	0.08 (−0.15 to 0.30)	0.50
Long-chain omega 3 fatty acids, mg/day	6.90 (−7.54 to 21.34)	0.34
EPA, mg/day	2.75 (−1.07 to 6.57)	0.16
DHA, mg/day	6.85 (−1.53 to 15.22)	0.11
Carbohydrates, g/day	−0.17 (−1.68 to 1.33)	0.82
Sugar, g/day	0.12 (−1.05 to 1.28)	0.71
Alcohol, g/day	−0.58 (−1.26 to 0.10)	0.09
Fibre, g/day	−0.66 (−0.48 to 0.35)	0.75
Caffeine, mg/day	−12.99 (−26.07 to 0.09)	0.051
Water, g/day	−6.91 (−55.18 to 41.36)	0.78
Cholesterol, mg/day	1.20 (−4.25 to 6.65)	0.66
Calcium, mg/day	6.33 (−5.69 to 18.35)	0.30
Folate, μg/day	3.34 (−4.49 to 11.17)	0.40
Iron, mg/day	−0.03 (−0.15 to 0.08)	0.57
Magnesium, mg/day	−5.46 (−11.42 to 0.50)	0.07
Sodium, mg/day	8.08 (−10.93 to 27.09)	0.40
Vitamin B12, mg/day	0.05 (−0.02 to 0.11)	0.14
Vitamin D, μg/day	−0.01 (−0.12 to 0.10)	0.88
Zinc, mg/day	0.08 (−0.03 to 0.18)	0.16
HEIFA-2013 total	0.06 (−0.58 to 0.70)	0.85

Y-BOCS, Yale–Brown Obsessive–Compulsive Scale; EPA, eicosapentaenoic acid; DHA, docosahexaenoic acid; HEIFA-2013, Healthy Eating Index for Australian Adults – 2013.

a. Adjusted for gender, age and energy intake

b. Per unit increase in total Y-BOCS score.

There was also some weak albeit non-significant evidence demonstrating that the relationship between OCD severity and magnesium intake varied with gender (interaction estimate of 13.74 mg/day, 95% CI −2.55 to 30.0, *P* = 0.097). In men, greater OCD severity was associated with greater magnesium intake (interaction estimate of 7.32 mg/day, 95% CI −6.85 to 21.50, *P* = 0.298), whereas in women, greater OCD severity was associated with lower magnesium intake (interaction estimate of −6.15 mg/day, 95% CI −15.45 to 3.15, *P* = 0.191), although non-significantly in each case. There was no evidence that the relationship between OCD severity and zinc intake differed by gender (interaction estimate of 0.19 mg/day, 95% CI −0.18 to 0.56, *P* = 0.317).

No substantive changes were seen in sensitivity analyses that included depression diagnosis as a covariate (Supplementary Table 1 available at https://doi.org/10.1192/bjo.2021.1039). In a further sensitivity analysis, including age, gender, total energy intake and treatment resistance as covariates, we found a statistically significant inverse association between OCD severity and caffeine (β = −15.50, 95% CI −28.88 to −2.11, *P* = 0.024), as well as an inverse association between OCD severity and magnesium (β = −6.63, 95% CI −12.72 to −0.53, *P* = 0.034). We additionally assessed whether OCD chronicity (years since diagnosis) was associated with nutrient intakes by using a further regression model, adjusted for age, gender, total energy intake and total Y-BOCS score. However, there was no evidence that OCD chronicity was associated with intakes of any nutrient (all *P* > 0.05; results not shown).

## Discussion

This study assessed dietary quality and nutrient intake in a sample of adults with OCD. Macronutrient intake in this sample largely aligned with Australian dietary guidelines, whereas dietary quality scores were higher than in previous studies of healthy samples. There was no significant association nutrient intake and dietary quality variables with OCD severity when adjusted for age, gender and total energy intake. However, in a sensitivity analysis which additionally included OCD treatment resistance as a covariate, OCD severity was inversely associated with caffeine (*P* = 0.024) and magnesium (*P* = 0.034) intake.

Average daily intake levels of macronutrients and micronutrients were largely in line with the 2013 Australian Dietary Guidelines, except for sodium and energy intake in men. Counter to our hypothesis, compared with a randomly sampled Australian population that were surveyed with a similar version of the DQES (3.0 and 3.1), the adult OCD sample in the present study had lower average daily intakes of total energy and macronutrients (but reported a higher consumption of alcohol; 7.93 g/day in this sample compared with 3.6 g/day in the other sample).^38^

In previous research evaluating dietary intake in individuals with schizophrenia and major depressive disorder, mean total energy and carbohydrate intake levels were higher in most of these samples, along with their respective control groups, than the OCD sample in the present study.^39–41^ Our OCD sample reported higher levels of monounsaturated fat intake than studies evaluating individuals with schizophrenia, bipolar disorder and major depressive disorder.^39,42^ These findings may be explained by the predominance of women in our sample, who have lower caloric intakes than men. However, it is difficult to draw direct conclusions when evaluating these studies, given the different measures of dietary intake and study locations.

The mean dietary quality in this sample was 59.9 (s.d. = 13.7) out of a total maximum score of 100. This finding was unexpectedly high, given that three other studies using the HEIFA-2013 in non-clinical samples reported mean dietary quality scores of between 45.5 and 53.84.^32,43,44^ There are a few potential reasons why the mean dietary quality in this sample may have been higher than in previous studies. Roy et al used a weighted food record, which is a more accurate measure of dietary intake.^44^ Conversely, FFQs have been found to overreport intakes of healthy food and underreport intakes of unhealthy foods, meaning that the methods used in the present study may have overestimated the HEIFA-2013 score.^45^ Second, the age ranges of two of the studies were exclusively young adults and university students, respectively, and thus are limited in comparability.^32,44^ Finally, there may be a genuine difference between patients with OCD and healthy controls, given the lower energy, protein and carbohydrate intakes compared with the two healthy control groups described earlier. It may be hypothesised that the large diagnostic comorbidity between OCD, anorexia nervosa and bulimia nervosa (because of the trait commonality of perfectionism and cognitive rigidity in these disorders) may contribute to these findings.^46^ However, we did not have sufficient data to examine this, and further research is required to explore this hypothesis.

We did not find a statistically significant correlation between total energy and macronutrient intake with OCD severity in this study, and therefore our hypothesis that OCD severity is associated with a higher macronutrient intake was not supported. One potential reason for the null findings could be related to selection bias. Participants with more severe OCD were relatively less likely to complete the DQES, and these participants might additionally have had poorer dietary intake. It is noteworthy that our positive albeit non-significant associations between total energy and macronutrient intake with OCD severity broadly aligns with a large population-scale analysis that found that people with more severe mental illness (i.e. major depressive disorder, schizophrenia and bipolar disorder) reported higher caloric and macronutrient intake compared with healthy controls.^47^ Therefore, further research in this area is warranted, given our findings were inconclusive.

Regression modelling identified an inverse association between OCD severity and caffeine consumption, when adjusting for age, gender, total energy intake and treatment resistance. Although this result is preliminary and has not been adjusted for multiple tests, this result is consistent with emerging data from two short-term RCTs (5 weeks, *n* = 24, dextroamphetamine versus caffeine; 8 weeks, *n* = 62, caffeine versus placebo) that reported significant reductions in OCD symptoms when caffeine was augmented in adults with treatment-resistant OCD.^48,49^ Additionally, in our recently completed open-label Treatment of Refractory Obsessive-Compulsive Disorder with Nutraceuticals (TRON) trial, we found that treatment response to a nutraceutical combination was substantially higher in participants with high levels of caffeine intake relative to those with low caffeine intake in a treatment resistant OCD sample.^26^ It has been hypothesised that caffeine's modulation of tryptophan, serotonin and noradrenaline production via adenosine A1 and A2 receptor antagonism is also implicated in the pathophysiology of OCD.^49^ However, larger samples and longer-term follow-up trials are required to determine whether a dose–response relationship exists.

In the regression models that included covariates for age, gender, total energy intake and OCD treatment resistance, an inverse association between magnesium intake and OCD severity was also noted. However, one other case–control study found no correlation between total Y-BOCS score and serum magnesium in a sample of 48 adults with OCD.^18^ However, comparisons with this study are limited by different study locations, gender ratios and methods of measuring magnesium levels. Given glutamate dysfunction has been implicated in the neurobiology of OCD and magnesium is linked with glutaminergic signal modulation, further trials elucidating this relationship in large clinical samples are warranted.^4,50^

Linear regression modelling also demonstrated tentative evidence that the relationship between OCD severity and magnesium intake may vary with gender, with inverse associations in women and positive associations in men (albeit non-significantly). These findings are noteworthy, given that two large population-based studies found significant, inverse associations between depressive symptoms and dietary magnesium intake in women, but not men.^22,23^ Larger samples of OCD cohorts with matched control groups are needed to elucidate these preliminary findings further.

Our findings are in partial agreement with the small body of literature looking at micronutrients and trace elements in relation to OCD. We found no significant associations between OCD severity and calcium, vitamin D, iron, zinc and folate intake, which largely aligns with previous case–control studies comparing these micronutrients with individuals with OCD.^18–20^ However, a study using a small sample of 23 adult patients with OCD found a significant negative relationship between serum folate and total Y-BOCS scores.^16^ In contrast with our findings, another case–control study found a negative correlation between total Y-BOCS scores and serum vitamin B12 and vitamin D in 52 children and adolescents with OCD.^17^ Given that these studies predominantly used serum level measurements of these micronutrients and were conducted in different study locations as well as contexts, comparability may be limited.

This study has many strengths. It is the first to describe in detail the dietary quality and nutrient intake in an adult sample with OCD. We were also able to compare dietary quality and nutrient intake to OCD severity, controlling for multiple confounding variables. Lastly, we were able to collect data from participants across multiple study sites. However, this study is not without its limitations. We used a cross-sectional data-set, which does not longitudinally assess dietary quality in relationship to OCD symptoms nor does it allow an assessment of causation. This study also lacks a control group with an identical FFQ to make proper comparisons using quantitative methods, as we were unable to locate data with the same questionnaire. The DQES version 3.2 also relies on recalling what foods were eaten in the past, which is subject to measurement bias and is likely to have underestimated nutrient intakes and overestimated the dietary quality scores. It is also unclear what role the presence of OCD might have on reporting of dietary intake. Finally, our sample size was modest, and we did not have enough participants across all obsessive–compulsive-related disorders to account for these potential confounders. Future research should be longitudinal in nature, use a larger sample size as well as weighted food records as a more accurate measure of nutrient intake, and employ a control group to make between-group comparisons.

In conclusion, the nutrient intake in our sample of patients with OCD largely aligned with recommended Australian dietary guidelines. Dietary quality was comparable with healthy samples, but limitations must be noted. Although OCD severity was inversely associated with caffeine and magnesium intake in one sensitivity analysis accounting for OCD treatment resistance, our findings largely showed that OCD severity had a minimal effect on nutrient intake and dietary quality. Future studies using a larger sample size and a control group will help better elucidate the relationship between nutrient intake and dietary quality in patients with OCD.

## Data Availability

The data that support the findings of this study are available from the corresponding author, J.S., upon reasonable request.

## Supplementary material

For supplementary material accompanying this paper visit http://dx.doi.org/10.1192/bjo.2021.1039. click here to view supplementary material

## References

[1] Brakoulias V, Starcevic V, Belloch A, Brown C, Ferrao YA, Fontenelle LF, Comorbidity, age of onset and suicidality in obsessive–compulsive disorder (OCD): an international collaboration. Compr Psychiatry 2017; 76: 79–86. 28433854 10.1016/j.comppsych.2017.04.002

[2] Koran LM, Hanna GL, Hollander E, Nestadt G, Simpson HB. Practice guideline for the treatment of patients with obsessive-compulsive disorder. Am J Psychiatry 2007; 164(suppl 7): 5–53. 17849776

[3] Pallanti S, Hollander E, Bienstock C, Koran L, Leckman J, Marazziti D, Treatment non-response in OCD: methodological issues and operational definitions. Int J Neuropsychopharmacol 2002; 5(2): 181–91. 12135542 10.1017/S1461145702002900

[4] Maia A, Oliveira J, Lajnef M, Mallet L, Tamouza R, Leboyer M, Oxidative and nitrosative stress markers in obsessive–compulsive disorder: a systematic review and meta-analysis. Acta Psychiatr Scand 2019; 139(5): 420–33. 30873609 10.1111/acps.13026

[5] Orhan N, Kucukali CI, Cakir U, Seker N, Aydin M. Genetic variants in nuclear-encoded mitochondrial proteins are associated with oxidative stress in obsessive compulsive disorders. J Psychiatr Res 2012; 46(2): 212–8. 22070905 10.1016/j.jpsychires.2011.09.012

[6] Turna J, Grosman Kaplan K, Anglin R, Patterson B, Soreni N, Bercik P, The gut microbiome and inflammation in obsessive-compulsive disorder patients compared to age- and sex-matched controls: a pilot study. Acta Psychiatr Scand 2020; 142(4): 337–47. 32307692 10.1111/acps.13175

[7] Godos J, Currenti W, Angelino D, Mena P, Castellano S, Caraci F, Diet and mental health: review of the recent updates on molecular mechanisms. Antioxidants (Basel) 2020; 9(4): 346. 10.3390/antiox9040346 PMC7222344 32340112

[8] Marx W, Lane M, Hockey M, Aslam H, Berk M, Walder K, Diet and depression: exploring the biological mechanisms of action. Mol Psychiatry 2021; 26(1): 134–50. 33144709 10.1038/s41380-020-00925-x

[9] Firth J, Marx W, Dash S, Carney R, Teasdale SB, Solmi M, The effects of dietary improvement on symptoms of depression and anxiety: a meta-analysis of randomized controlled trials. Psychosom Med 2019; 81(3): 265–80. 30720698 10.1097/PSY.0000000000000673 PMC6455094

[10] Lassale C, Batty GD, Baghdadli A, Jacka F, Sánchez-Villegas A, Kivimäki M, Healthy dietary indices and risk of depressive outcomes: a systematic review and meta-analysis of observational studies. Mol Psychiatry 2019; 24(7): 965–86. 30254236 10.1038/s41380-018-0237-8 PMC6755986

[11] Firth J, Solmi M, Wootton RE, Vancampfort D, Schuch FB, Hoare E, A meta-review of “lifestyle psychiatry”: the role of exercise, smoking, diet and sleep in the prevention and treatment of mental disorders. World Psychiatry 2020; 19(3): 360–80. 32931092 10.1002/wps.20773 PMC7491615

[12] Isomura K, Brander G, Chang Z, Kuja-Halkola R, Rück C, Hellner C, Metabolic and cardiovascular complications in obsessive-compulsive disorder: a total population, sibling comparison study with long-term follow-up. Biol Psychiatry 2018; 84(5): 324–31. 29395042 10.1016/j.biopsych.2017.12.003

[13] Albert U, Aguglia A, Chiarle A, Bogetto F, Maina G. Metabolic syndrome and obsessive–compulsive disorder: a naturalistic Italian study. Gen Hosp Psychiatry 2013; 35(2): 154–9. 23158675 10.1016/j.genhosppsych.2012.10.004

[14] Drummond LM, Boschen MJ, Cullimore J, Khan-Hameed A, White S, Ion R. Physical complications of severe, chronic obsessive-compulsive disorder: a comparison with general psychiatric inpatients. Gen Hosp Psychiatry 2012; 34(6): 618–25. 22459999 10.1016/j.genhosppsych.2012.02.001

[15] Teasdale SB, Ward PB, Samaras K, Firth J, Stubbs B, Tripodi E, Dietary intake of people with severe mental illness: systematic review and meta-analysis. Br J Psychiatry 2019; 214(5): 251–9. 30784395 10.1192/bjp.2019.20

[16] Atmaca M, Tezcan E, Kuloglu M, Kirtas O, Ustundag B. Serum folate and homocysteine levels in patients with obsessive-compulsive disorder. Psychiatry Clin Neurosci 2005; 59(5): 616–20. 16194269 10.1111/j.1440-1819.2005.01425.x

[17] Esnafoğlu E, Yaman E. Vitamin B12, folic acid, homocysteine and vitamin D levels in children and adolescents with obsessive compulsive disorder. Psychiatry Res 2017; 254: 232–7. 28477545 10.1016/j.psychres.2017.04.032

[18] Shohag H, Ullah A, Qusar S, Rahman M, Hasnat A. Alterations of serum zinc, copper, manganese, iron, calcium, and magnesium concentrations and the complexity of interelement relations in patients with obsessive–compulsive disorder. Biol Trace Elem Res 2012; 148(3): 275–80. 22383079 10.1007/s12011-012-9371-3

[19] Türksoy N, Bilici R, Yalçıner A, Ozdemir YÖ, Ornek I, Tufan AE, Vitamin B12, folate, and homocysteine levels in patients with obsessive-compulsive disorder. Neuropsychiatr Dis Treat 2014; 10: 1671–5. 25228807 10.2147/NDT.S67668 PMC4164291

[20] Yazici KU, Percinel Yazici I, Ustundag B. Vitamin D levels in children and adolescents with obsessive compulsive disorder. Nord J Psychiatry 2018; 72(3): 173–8. 29168423 10.1080/08039488.2017.1406985

[21] Maserejian NN, Hall SA, McKinlay JB. Low dietary or supplemental zinc is associated with depression symptoms among women, but not men, in a population-based epidemiological survey. J Affect Disord 2012; 136(3): 781–8. 22030131 10.1016/j.jad.2011.09.039 PMC3272121

[22] Sun C, Wang R, Li Z, Zhang D. Dietary magnesium intake and risk of depression. J Affect Disord 2019; 246: 627–32. 30611059 10.1016/j.jad.2018.12.114

[23] Thi Thu Nguyen T, Miyagi S, Tsujiguchi H, Kambayashi Y, Hara A, Nakamura H, Association between lower intake of minerals and depressive symptoms among elderly Japanese women but not men: findings from Shika study. Nutrients 2019; 11(2): 389. 10.3390/nu11020389 PMC6412241 30781841

[24] Sayyah M, Olapour A, Saeedabad Y, Yazdan Parast R, Malayeri A. Evaluation of oral zinc sulfate effect on obsessive-compulsive disorder: a randomized placebo-controlled clinical trial. Nutrition 2012; 28(9): 892–5. 22465904 10.1016/j.nut.2011.11.027

[25] Australian and New Zealand Clinical Trials Registry. N-Acetyl Cysteine (NAC) Augmentation in Obsessive-Compulsive Disorder (OCD): A 24-Week, Randomized, Double Blind Placebo Controlled Trial. Australian and New Zealand Clinical Trials Registry, 2016 (https://www.anzctr.org.au/Trial/Registration/TrialReview.aspx?id=370954).

[26] Sarris J, Byrne GJ, Oliver G, Cribb L, Blair-West S, Castle D, Treatment of refractory obsessive–compulsive disorder with nutraceuticals (TRON): a 20-week, open label pilot study. CNS Spectrums [Epub ahead of print] 21 Jun 2021. Available from: 10.1017/S1092852921000638. 34165060

[27] Goodman WK, Price LH, Rasmussen SA, Mazure C, Delgado P, Heninger GR, The Yale-Brown Obsessive Compulsive Scale: II. Validity. Arch Gen Psychiatry 1989; 46(11): 1012–6. 2510699 10.1001/archpsyc.1989.01810110054008

[28] Goodman WK, Price LH, Rasmussen SA, Mazure C, Fleischmann RL, Hill CL, The Yale-Brown Obsessive Compulsive Scale: I. Development, use, and reliability. Arch Gen Psychiatry 1989; 46(11): 1006–11. 2684084 10.1001/archpsyc.1989.01810110048007

[29] Grabill K, Merlo L, Duke D, Harford K-L, Keeley ML, Geffken GR, Assessment of obsessive–compulsive disorder: a review. J Anxiety Disord 2008; 22(1): 1–17. 17367988 10.1016/j.janxdis.2007.01.012

[30] Bassett JK, English DR, Fahey MT, Forbes AB, Gurrin LC, Simpson JA, Validity and calibration of the FFQ used in the Melbourne Collaborative Cohort Study. Public Health Nutr 2016; 19(13): 2357–68. 27075344 10.1017/S1368980016000690 PMC10271053

[31] Zhang AC, Downie LE. Preliminary validation of a food frequency questionnaire to assess long-chain omega-3 atty acid intake in eye care practice. Nutrients 2019; 11(4): 817. 10.3390/nu11040817 PMC6521311 30978959

[32] Roy R, Hebden L, Rangan A, Allman-Farinelli M. The development, application, and validation of a Healthy Eating Index for Australian Adults (HEIFA—2013). Nutrition 2016; 32(4): 432–40. 26740257 10.1016/j.nut.2015.10.006

[33] Hlaing-Hlaing H, Pezdirc K, Tavener M, James EL, Hure A. Diet quality indices used in Australian and New Zealand adults: a systematic review and critical appraisal. Nutrients 2020; 12(12): 3777. 10.3390/nu12123777 PMC7763901 33317123

[34] Buuren SV, Groothuis-Oudshoorn K. mice: Multivariate imputation by chained equations in R. J Stat Softw 2010; 45(3): 1–67.

[35] National Health and Medical Research Council. Australian Dietary Guidelines. National Health and Medical Research Council, 2013 (https://www.eatforhealth.gov.au/sites/default/files/files/the_guidelines/n55_australian_dietary_guidelines.pdf).

[36] National Health and Medical Research Council, Australian Government Department of Health and Ageing, New Zealand Ministry of Health. Nutrient Reference Values for Australia and New Zealand. National Health and Medical Research Council, 2006 (https://www.nhmrc.gov.au/sites/default/files/images/nutrient-refererence-dietary-intakes.pdf).

[37] Bracci EL, Keogh JB, Milte R, Murphy KJ. A comparison of dietary quality and nutritional adequacy of popular energy-restricted diets against the Australian guide to healthy eating and the Mediterranean diet. Br J Nutr [Epub ahead of print] 21 Jun 2021. Available from: 10.1017/S0007114521002282. 34155964

[38] Fulton AS, Baldock KL, Coates AM, Williams MT, Howe PRC, Haren MT, Polyunsaturated fatty acid intake and lung function in a regional Australian population: a cross-sectional study with a nested case-control analysis. J Nutr Int Metab 2019; 18: 100102.

[39] Jahrami H, Saif Z, AlHaddad M, MeA-I F, Hammad L, Ali B. Assessing dietary and lifestyle risk behaviours and their associations with disease comorbidities among patients with depression: a case-control study from Bahrain. Heliyon 2020; 6(6): e04323. 32637706 10.1016/j.heliyon.2020.e04323 PMC7327261

[40] Nunes D, Eskinazi B, Camboim Rockett F, Delgado VB, Schweigert Perry ID. Nutritional status, food intake and cardiovascular disease risk in individuals with schizophrenia in southern Brazil: a case–control study. Rev Psiquiatr Salud Ment 2014; 7(2): 72–9. 24054065 10.1016/j.rpsm.2013.07.001

[41] Strassnig M, Brar JS, Ganguli R. Dietary intake of patients with schizophrenia. Psychiatry (Edgmont) 2005; 2(2): 31–5. PMC3004718 21179633

[42] Bly MJ, Taylor SF, Dalack G, Pop-Busui R, Burghardt KJ, Evans SJ, Metabolic syndrome in bipolar disorder and schizophrenia: dietary and lifestyle factors compared to the general population. Bipolar Disord 2014; 16(3): 277–88. 24330321 10.1111/bdi.12160 PMC4023536

[43] Grech A, Sui Z, Siu HY, Zheng M, Allman-Farinelli M, Rangan A. Socio-demographic determinants of diet quality in Australian adults using the validated healthy eating index for Australian adults (HEIFA-2013). Healthcare (Basel) 2017; 5(1): 7. 10.3390/healthcare5010007 PMC5371913 28165394

[44] Roy R, Rangan A, Hebden L, Yu Louie JC, Tang LM, Kay J, Dietary contribution of foods and beverages sold within a university campus and its effect on diet quality of young adults. Nutrition 2017; 34: 118–23. 28063506 10.1016/j.nut.2016.09.013

[45] Johansson I, Hallmans G, Wikman Å, Biessy C, Riboli E, Kaaks R. Validation and calibration of food-frequency questionnaire measurements in the Northern Sweden Health and Disease cohort. Public Health Nutr 2002; 5(3): 487–96. 12003662 10.1079/phn2001315

[46] Jiménez-Murcia S, Fernández-Aranda F, Raich RM, Alonso P, Krug I, Jaurrieta N, Obsessive-compulsive and eating disorders: comparison of clinical and personality features. Psychiatry Clin Neurosci 2007; 61(4): 385–91. 17610663 10.1111/j.1440-1819.2007.01673.x

[47] Firth J, Stubbs B, Teasdale SB, Ward PB, Veronese N, Shivappa N, Diet as a hot topic in psychiatry: a population-scale study of nutritional intake and inflammatory potential in severe mental illness. World Psychiatry 2018; 17(3): 365–7. 30192082 10.1002/wps.20571 PMC6127755

[48] Koran LM, Aboujaoude E, Gamel NN. Double-blind study of dextroamphetamine versus caffeine augmentation for treatment-resistant obsessive-compulsive disorder. J Clin Psychiatry 2009; 70(11): 1530–5. 19573497 10.4088/JCP.08m04605

[49] Shams J, Soufi ES, Zahiroddin A, Shekarriz-Foumani R. Using caffeine on the patients as therapeutic option against treatment-resistant obsessive-compulsive disorder. J Family Med Prim Care 2019; 8(5): 1741–7. 31198747 10.4103/jfmpc.jfmpc_93_19 PMC6559101

[50] Botturi A, Ciappolino V, Delvecchio G, Boscutti A, Viscardi B, Brambilla P. The role and the effect of magnesium in mental disorders: a systematic review. Nutrients 2020; 12(6): 1661. 10.3390/nu12061661 PMC7352515 32503201

