# Supplementary material for: Dietary quality and nutrient intake in adults with obsessive–compulsive disorder
Source: BJPsych Open. 2021 Nov 19;7(6):e218. doi: 10.1192/bjo.2021.1039 (PMC8612013; doi:10.1192/bjo.2021.1039)
Supplement: Supplementary file 1 [file bjosup.zip › S2056472421010395sup001.docx]

Table 1. Participant characteristics

| *Characteristic* | *Summary* |
| --- | --- |
| Age (years), mean (SD) | 36.95 (14.3) |
| Gender |  |
| Male, n (%) | 28 (32.9) |
| Female, n (%) | 57 (67.1) |
| BMI, mean (SD) | 27.14 (6.3) |
| Marital status, n (%) |  |
| Single | 42 (49.4) |
| Married/defacto | 32 (37.6) |
| Separated/divorced/widowed | 7 (28.2) |
| In a relationship | 4 (4.7) |
| Education, n (%) |  |
| High school graduate or early leaver | 29 (34.1) |
| College certificate/diploma | 18 (21.2) |
| University graduate/post-graduate | 38 (44.7) |
| Employment status, n (%) |  |
| Employed | 46 (54.1) |
| Student | 14 (16.5) |
| Unemployed | 25 (29.4) |
| Trial, n (%) |  |
| NAC | 67 (78.8) |
| TRON | 18 (21.2) |
| Trial site, n (%) |  |
| Melbourne | 43 (50.6) |
| Brisbane | 30 (35.3) |
| Sydney | 12 (14.1) |

BMI = body mass index, NAC = n-acetyl cysteine, TRON = Treatment of Refractory Obsessive-Compulsive Disorder with Nutraceuticals.

Table 2. Participant psychological features

| *Psychological characteristic* | *Summary* |
| --- | --- |
| Age onset of OCD symptoms (years), mean (SD)^1^ | 13.4 (9.8) |
| Duration of OCD symptoms (years), mean (SD)^1^ | 23.3 (12.2) |
| Age of OCD diagnosis (years), mean (SD) | 23.85 (10.8) |
| Chronicity of OCD diagnosis (years), mean (SD) | 13.15 (10.7) |
| Number of unsuccessful prior medication & therapy trials, mean (SD)^1^ | 2.66 (2.9) |
| Treatment resistance, n (%) |  |
| Yes | 18 (21.2) |
| No | 67 (78.8) |
| YBOCS total, mean (SD) | 22.7 (4.5) |
| Moderate, n (%) | 52 (61.2) |
| Severe, n (%) | 31 (36.5) |
| Extreme, n (%) | 2 (2.4) |
| DOCS total, mean (SD)^2^ | 29.9 (13.2) |
| BAI total, mean (SD) | 18.1 (11.8) |
| SIGHD total, mean (SD) | 10.0 (6.3) |
| SDS total, mean (SD) | 13.4 (6.9) |
| WHOQOL-BREF total, mean (SD)^3^ | 80.6 (13.5) |
| Patient global impression, mean (SD)^4^ | 2.9 (0.6) |
| Clinician global impression, mean (SD) | 4.1 (0.8) |
| Current major depressive episode, n (%) | 14 (16.5) |
| Previous major depressive episodes, n (%)^1^ | 53 (62.4) |
| Diagnosis of generalized anxiety disorder, n (%)^3^ | 19 (23.2) |
| Diagnosis of panic disorder, n (%)^5^ | 13 (16) |
| Diagnosis of social anxiety disorder, n (%)^6^ | 15 (19) |
| Diagnosis of excoriation disorder, n (%)^5^ | 8 (9.9) |

BAI = Beck Anxiety Inventory; DOCS = Dimensional Obsessive-Compulsive Scale; SDS = The Sheehan Disability Scale; SIGHD = Structured Interview Guide for the Hamilton Depression Rating Scale; WHOQOL-BREF = World Health Organization Quality of Life **–** BREF; YBOCS = Yale-Brown Obsessive-Compulsive Scale

^1^ n=2 missing, ^2^ n=8 missing, ^3^ n=3 missing, ^4^ n=1 missing, ^5^ n=4 missing, ^6^ n=6 missing

Table 3. Participant dietary intake and quality with Australian recommended intake levels

| *Dietary characteristic* | *Overall, mean (SD)* | *Male, mean (SD)* | *Female, mean (SD)* | *Upper limit* | *Recommended intake for 31–50-year-olds^*^* | |
| --- | --- | --- | --- | --- | --- | --- |
|  |  |  |  |  | *Males* | *Females* |
| Energy (kJ/day) | 8297.94 (2628.1) | 9439.47 (2697.85) | 7737.18 (2424.71) | - | 8700 (dependent on BMI, physical activity level and age) | |
| Protein (g/day) | 84.78 (32.1) | 99.07 (33.84) | 77.76 (29.05) | - | 64 | 46 |
| Fat (g/day) | 84.16 (27.5) | 92.64 (29.77) | 80.00 (25.55) | - | - | - |
| Saturated fatty acids (g/day) | 27.83 (10.8) | 31.33 (10.83) | 26.11 (10.40) | - | - | - |
| Monounsaturated fatty acids (g/day) | 35.46 (12.1) | 39.34 (13.86) | 33.55 (10.69) | - | - | - |
| Polyunsaturated fatty acids (g/day) | 14.93 (6.3) | 15.71 (6.72) | 14.54 (6.03) | - | 13~ | 8~ |
| Long chain omega 3 fatty acids (mg/day) | 343.16 (313.2) | 449.17 (363.76) | 291.09 (273.86) | - | - | - |
| Eicosapentaenoic acid – EPA (mg/day) | 88.26 (84.1) | 119.33 (102.75) | 72.99 (69.36) | - | - | - |
| Docosahexaenoic acid – DHA (mg/day) | 176.70 (183.1) | 244.04 (232.65) | 143.62 (144.30) | - | - | - |
| Carbohydrates (g/day) | 189.06 (67.2) | 214.92 (65.99) | 176.35 (64.63) | - | - | - |
| Sugar (g/day) | 95.31 (37.6) | 106.91 (33.54) | 89.61 (38.44) | - | - | - |
| Alcohol (g/day) | 7.93 (14.2) | 10.79 (13.58) | 6.52 (14.44) | M=40, F=20 | - | - |
| Fibre (g/day) | 25.43 (11.8) | 26.24 (13.23) | 25.03 (11.10) | - | 30^ | 25^ |
| Caffeine (mg/day) | 278.28 (278.8) | 366.66 (353.33) | 234.86 (224.92) | 400 | N/A | N/A |
| Water (g/day) | 4924.78 (1059.7) | 3333.69 (1103.52) | 2849.84 (1009.44) | - | 3400^ | 2800^ |
| Cholesterol (mg/day) | 274.36 (124.3) | 301.19 (114.21) | 261.17 (127.91) | - | - | - |
| Calcium (mg/day) | 865.88 (372.2) | 1001.66 (353.15) | 799.33 (366.01) | 2500 | 1000 | 1000 |
| Folate (ug/day) | 420.41 (194.1) | 446.06 (221.50) | 407.81 (179.83) | 1000 | 400 | 400 |
| Iron (mg/day) | 10.96 (3.8) | 11.69 (4.46) | 10.61 (3.39) | 45 | 8 | 18 |
| Magnesium (mg/day) | 430.2 (164.7) | 493.00 (165.96) | 399.28 (156.43) | 350 | 420 | 320 |
| Sodium (mg/day) | 2172.13 (805.5) | 2556.05 (920.82) | 1983.55 (674.27) | 2300 | 460-920^ | 460-620^ |
| Vitamin B12 (mg/day) | 3.13 (1.7) | 4.20 (1.9) | 2.61 (1.37) | 80 | 2.4 | 2.4 |
| Vitamin D (ug/day) | 3.72 (2.6) | 5.19 (2.9) | 3.00 (2.11) | 80 | 5^ | 5^ |
| Zinc (mg/day) | 8.84 (3.8) | 9.96 (4.3) | 8.29 (3.34) | 40 | 14 | 8 |
| HEIFA total | 59.9 (13.7) | 56.2 (14.5) | 58.4 (12.2) | 100 | - | - |
| Discretionary | 5.2 (3.9) | 4.6 (4.2) | 5.5 (3.7) | 10 | - | - |
| Vegetables | 4.6 (2.8) | 3.7 (2.7) | 5.1 (2.8) | 10 | - | - |
| Fruit | 8.0 (1.9) | 7.8 (2.3) | 8.1 (1.7) | 10 | - | - |
| Grains | 3.9 (3.1) | 5.0 (3.4) | 3.5 (2.9) | 10 | - | - |
| Protein | 7.9 (2.9) | 7.7 (2.9) | 8.1 (2.9) | 10 | - | - |
| Dairy | 5.2 (3.5) | 7.1 (3.3) | 4.3 (3.2) | 10 | - | - |
| Fluid | 3.6 (1.6) | 3.3 (1.7) | 3.7 (1.5) | 5 | - | - |
| Fat | 6.3 (2.0) | 6.3 (2.1) | 6.4 (1.9) | 10 | - | - |
| Sodium | 4.5 (4.0) | 3.2 (3.7) | 5.1 (4.1) | 10 | - | - |
| Sugar | 6.3 (4.2) | 5.7 (4.5) | 6.6 (4.0) | 10 | - | - |
| Alcohol | 4.3 (1.8) | 3.8 (2.2) | 4.6 (1.4) | 5 | - | - |

^*^ Recommended daily intake (RDI) unless otherwise stated; ^Adequate intake, ~Linoleic acid, M = Male, F = Female; Recommended daily intakes are taken from the Nutrient Reference Values for Australian and New Zealand (44).

Table 4. Associations between total YBOCS score and dietary intake/quality

|  | Model 1^1^ | |
| --- | --- | --- |
| *Dietary characteristic* | *Adjusted coefficient (95% CI)** | *P-value* |
| Protein (g/day) | 0.35 (-0.43 – 1.13) | 0.37 |
| Fat (g/day) | 0.45 (-0.16 – 1.06) | 0.15 |
| Saturated fatty acids (g/day) | 0.22 (-0.07 – 0.50) | 0.14 |
| Monounsaturated fatty acids (g/day) | 0.14 (-0.22 – 0.51) | 0.43 |
| Polyunsaturated fatty acids (g/day) | 0.08 (-0.15 – 0.30) | 0.50 |
| Long chain omega 3 fatty acids (mg/day) | 6.90 (-7.54 – 21.34) | 0.34 |
| EPA (mg/day) | 2.75 (-1.07 – 6.57) | 0.16 |
| DHA (mg/day) | 6.85 (-1.53 – 15.22) | 0.11 |
| Carbohydrates (g/day) | -0.17 (-1.68 – 1.33) | 0.82 |
| Sugar (g/day) | 0.12 (-1.05 – 1.28) | 0.71 |
| Alcohol (g/day) | -0.58 (-1.26 – 0.10) | 0.09 |
| Fibre (g/day) | -0.66 (-0.48 – 0.35) | 0.75 |
| Caffeine (mg/day) | -12.99 (-26.07 – 0.09) | 0.051 |
| Water (g/day) | -6.91 (-55.18 – 41.36) | 0.78 |
| Cholesterol (mg/day) | 1.20 (-4.25 – 6.65) | 0.66 |
| Calcium (mg/day) | 6.33 (-5.69 – 18.35) | 0.30 |
| Folate (ug/day) | 3.34 (-4.49 – 11.17) | 0.40 |
| Iron (mg/day) | -0.03 (-0.15 – 0.08) | 0.57 |
| Magnesium (mg/day) | -5.46 (-11.42 – 0.50) | 0.07 |
| Sodium (mg/day) | 8.08 (-10.93 – 27.09) | 0.40 |
| Vitamin B12 (mg/day) | 0.05 (-0.02 – 0.11) | 0.14 |
| Vitamin D (ug/day) | -0.01 (-0.12 – 0.10) | 0.88 |
| Zinc (mg/day) | 0.08 (-0.03 – 0.18) | 0.16 |
| HEIFA total | 0.06 (-0.58 – 0.70) | 0.85 |

DHA = Docosahexaenoic acid, EPA = Eicosapentaenoic acid, HEIFA = Healthy Eating Index for Australian Adults

^*^ Per unit increase in total YBOCS score; ^1^Adjusted for gender, age and energy intake

**Supplementary Materials**

Table A. Associations between dietary intake/quality and total YBOCS score in sensitivity analysis including depression and treatment-resistance as covariates

|  | Model 2^1^ | | Model 3^2^ | |
| --- | --- | --- | --- | --- |
| *Dietary characteristic* | *Adjusted (95% CI)* | *Adjusted (95% CI)* | *Adjusted (95% CI)* | *P-value* |
| Protein (g/day) | 0.27 (-0.53 -1.08) | 0.27 (-0.53 -1.08) | 0.17 (-0.62 – 0.97) | 0.67 |
| Fat (g/day) | 0.35 (-0.28 – 0.97) | 0.35 (-0.28 – 0.97) | 0.42 (-0.22 – 1.05) | 0.20 |
| Saturated fatty acids (g/day) | 0.24 (-0.06 – 0.53) | 0.24 (-0.06 – 0.53) | 0.16 (-0.13 – 0.45) | 0.28 |
| Monounsaturated fatty acids (g/day) | 0.07 (-0.30 – 0.43) | 0.07 (-0.30 – 0.43) | 0.14 (-0.23 – 0.52) | 0.45 |
| Polyunsaturated fatty acids (g/day) | 0.04 (-0.19 – 0.27) | 0.04 (-0.19 – 0.27) | 0.10 (-0.13 – 0.34) | 0.38 |
| Long chain omega 3 fatty acids (mg/day) | 6.47 (-8.44 – 21.38) | 6.47 (-8.44 – 21.38) | 6.02 (-8.95 – 20.99) | 0.43 |
| EPA (mg/day) | 2.58 (-1.36 – 6.52) | 2.58 (-1.36 – 6.52) | 2.35 (-1.59 – 6.30) | 0.24 |
| DHA (mg/day) | 6.38 (-2.27 – 15.03) | 6.38 (-2.27 – 15.03) | 5.99 (-2.67 – 14.66) | 0.17 |
| Carbohydrates (g/day) | 0.18 (-1.33 – 1.70) | 0.18 (-1.33 – 1.70) | -0.02 (-1.58 – 1.54) | 0.98 |
| Sugar (g/day) | 0.27 (-0.93 – 1.46) | 0.27 (-0.93 – 1.46) | 0.19 (-1.02 – 1.39) | 0.76 |
| Alcohol (g/day) | -0.62 (-1.32 – 0.78) | -0.62 (-1.32 – 0.78) | -0.48 (-1.18 – 0.22) | 0.17 |
| Fibre (g/day) | -0.05 (-0.48 – 0.38) | -0.05 (-0.48 – 0.38) | -0.08 (-0.51 – 0.35) | 0.71 |
| Caffeine (mg/day) | -11.24 (-24.65 – 2.16) | -11.24 (-24.65 – 2.16) | -15.50 (-28.88 - -2.11) | 0.024* |
| Water (g/day) | -9.69 (-59.49 – 40.11) | -9.69 (-59.49 – 40.11) | -7.98 (-58.09 – 42.13) | 0.75 |
| Cholesterol (mg/day) | 0.52 (-5.07 – 6.11) | 0.52 (-5.07 – 6.11) | 0.93 (-4.72 – 6.59) | 0.74 |
| Calcium (mg/day) | 7.43 (-4.94 – 19.80) | 7.43 (-4.94 – 19.80) | 4.85 (-7.55 – 17.26) | 0.44 |
| Folate (ug/day) | 4.57 (-3.42 – 12.57) | 4.57 (-3.42 – 12.57) | 1.36 (-6.57 – 9.28) | 0.53 |
| Iron (mg/day) | -0.03 (-0.15 – 0.09) | -0.03 (-0.15 – 0.09) | -0.05 (-0.16 – 0.07) | 0.42 |
| Magnesium (mg/day) | -5.18 (-11.34 – 0.97) | -5.18 (-11.34 – 0.97) | -6.63 (-12.72 - -0.53) | 0.034* |
| Sodium (mg/day) | 8.45 (-11.19 – 28.09) | 8.45 (-11.19 – 28.09) | 3.32 (-15.93 – 22.58) | 0.73 |
| Vitamin B12 (mg/day) | 0.05 (-0.02 – 0.12) | 0.05 (-0.02 – 0.12) | 0.03 (-0.04 - 0.09) | 0.43 |
| Vitamin D (ug/day) | -0.004 (-0.12 – 0.11) | -0.004 (-0.12 – 0.11) | -0.04 (-0.15 – 0.08) | 0.53 |
| Zinc (mg/day) | 0.06 (-0.05 – 0.17) | 0.06 (-0.05 – 0.17) | 0.05 (-0.06 – 0.16) | 0.35 |
| HEIFA total | 0.02 (-0.64 – 0.68) | 0.02 (-0.64 – 0.68) | -0.02 (-0.68 – 0.64) | 0.95 |

DHA = Docosahexaenoic acid, EPA = Eicosapentaenoic acid, HEIFA = Healthy Eating Index for Australian Adults

^1^Adjusted for gender, age, depression and energy intake

^2^Adjusted for gender, age, treatment-resistance and energy intake

* *p* < 0.05
