# Supplementary material for: Dietary quality and nutrient intake in adults with obsessive–compulsive disorder
Source: BJPsych Open. 2021 Nov 19;7(6):e218. doi: 10.1192/bjo.2021.1039 (PMC8612013; doi:10.1192/bjo.2021.1039)
Supplement: Supplementary file 1 [file bjosup.zip › S2056472421010395sup002.docx]

**Supplementary Materials**

Table A. Associations between dietary intake/quality and total YBOCS score in sensitivity analysis including depression and treatment-resistance as covariates

|  | Model 2^1^ | | Model 3^2^ | |
| --- | --- | --- | --- | --- |
| *Dietary characteristic* | *Adjusted (95% CI)* | *Adjusted (95% CI)* | *Adjusted (95% CI)* | *P-value* |
| Protein (g/day) | 0.27 (-0.53 -1.08) | 0.27 (-0.53 -1.08) | 0.17 (-0.62 – 0.97) | 0.67 |
| Fat (g/day) | 0.35 (-0.28 – 0.97) | 0.35 (-0.28 – 0.97) | 0.42 (-0.22 – 1.05) | 0.20 |
| Saturated fatty acids (g/day) | 0.24 (-0.06 – 0.53) | 0.24 (-0.06 – 0.53) | 0.16 (-0.13 – 0.45) | 0.28 |
| Monounsaturated fatty acids (g/day) | 0.07 (-0.30 – 0.43) | 0.07 (-0.30 – 0.43) | 0.14 (-0.23 – 0.52) | 0.45 |
| Polyunsaturated fatty acids (g/day) | 0.04 (-0.19 – 0.27) | 0.04 (-0.19 – 0.27) | 0.10 (-0.13 – 0.34) | 0.38 |
| Long chain omega 3 fatty acids (mg/day) | 6.47 (-8.44 – 21.38) | 6.47 (-8.44 – 21.38) | 6.02 (-8.95 – 20.99) | 0.43 |
| EPA (mg/day) | 2.58 (-1.36 – 6.52) | 2.58 (-1.36 – 6.52) | 2.35 (-1.59 – 6.30) | 0.24 |
| DHA (mg/day) | 6.38 (-2.27 – 15.03) | 6.38 (-2.27 – 15.03) | 5.99 (-2.67 – 14.66) | 0.17 |
| Carbohydrates (g/day) | 0.18 (-1.33 – 1.70) | 0.18 (-1.33 – 1.70) | -0.02 (-1.58 – 1.54) | 0.98 |
| Sugar (g/day) | 0.27 (-0.93 – 1.46) | 0.27 (-0.93 – 1.46) | 0.19 (-1.02 – 1.39) | 0.76 |
| Alcohol (g/day) | -0.62 (-1.32 – 0.78) | -0.62 (-1.32 – 0.78) | -0.48 (-1.18 – 0.22) | 0.17 |
| Fibre (g/day) | -0.05 (-0.48 – 0.38) | -0.05 (-0.48 – 0.38) | -0.08 (-0.51 – 0.35) | 0.71 |
| Caffeine (mg/day) | -11.24 (-24.65 – 2.16) | -11.24 (-24.65 – 2.16) | -15.50 (-28.88 - -2.11) | 0.024* |
| Water (g/day) | -9.69 (-59.49 – 40.11) | -9.69 (-59.49 – 40.11) | -7.98 (-58.09 – 42.13) | 0.75 |
| Cholesterol (mg/day) | 0.52 (-5.07 – 6.11) | 0.52 (-5.07 – 6.11) | 0.93 (-4.72 – 6.59) | 0.74 |
| Calcium (mg/day) | 7.43 (-4.94 – 19.80) | 7.43 (-4.94 – 19.80) | 4.85 (-7.55 – 17.26) | 0.44 |
| Folate (ug/day) | 4.57 (-3.42 – 12.57) | 4.57 (-3.42 – 12.57) | 1.36 (-6.57 – 9.28) | 0.53 |
| Iron (mg/day) | -0.03 (-0.15 – 0.09) | -0.03 (-0.15 – 0.09) | -0.05 (-0.16 – 0.07) | 0.42 |
| Magnesium (mg/day) | -5.18 (-11.34 – 0.97) | -5.18 (-11.34 – 0.97) | -6.63 (-12.72 - -0.53) | 0.034* |
| Sodium (mg/day) | 8.45 (-11.19 – 28.09) | 8.45 (-11.19 – 28.09) | 3.32 (-15.93 – 22.58) | 0.73 |
| Vitamin B12 (mg/day) | 0.05 (-0.02 – 0.12) | 0.05 (-0.02 – 0.12) | 0.03 (-0.04 - 0.09) | 0.43 |
| Vitamin D (ug/day) | -0.004 (-0.12 – 0.11) | -0.004 (-0.12 – 0.11) | -0.04 (-0.15 – 0.08) | 0.53 |
| Zinc (mg/day) | 0.06 (-0.05 – 0.17) | 0.06 (-0.05 – 0.17) | 0.05 (-0.06 – 0.16) | 0.35 |
| HEIFA total | 0.02 (-0.64 – 0.68) | 0.02 (-0.64 – 0.68) | -0.02 (-0.68 – 0.64) | 0.95 |

DHA = Docosahexaenoic acid, EPA = Eicosapentaenoic acid, HEIFA = Healthy Eating Index for Australian Adults

^1^Adjusted for gender, age, depression and energy intake

^2^Adjusted for gender, age, treatment-resistance and energy intake

* *p* < 0.05
